# A home-based VR exercise for improving depression and quality of life after heart surgery: a randomized controlled trial

**DOI:** 10.1080/21642850.2026.2614807

**Published:** 2026-01-13

**Authors:** Kornanong Yuenyongchaiwat, Natsinee Sermsinsaithong, Chatchai Buekban, Chusak Thanawattano, Wararat Tavonudomgit, Chitima Kulchanarat, Khanistha Wattanananont, Sasipa Buranapuntalug, Preeyaphorn Songsorn, Opas Satdhabudha

**Affiliations:** a Department of Physical Therapy, Faculty of Allied Health Sciences, Thammasat University, Pathum Thani, Thailand; b Thammasat University Research Unit, Physical Therapy in Respiratory and Cardiovascular Systems, Thammasat University, Pathum Thani, Thailand; c Biomedical Electronics and Systems Research Team, Assistive Technology and Medical Devices Research Group, National Electronics and Computer Technology Center, Pathum Thani, Thailand; d Physical Therapy Center, Thammasat University Hospital, Pathum Thani, Thailand; e Cardiac Rehabilitation Unit, Excellence Center, Faculty of Medicine, Navamindradhiraj University, Bangkok, Thailand; f Department of Surgery, Faculty of Medicine, Thammasat University, Pathum Thani, Thailand

**Keywords:** Virtual reality, quality of life, heart surgery, depression, cardiac rehabilitation

## Abstract

**Background:**

Exercise is an integral component of cardiac rehabilitation after open-heart surgery in both hospital and community settings, and such programs are designed to reduce adverse physical and mental health outcomes in patients undergoing cardiac surgery. Virtual reality (VR)-based aerobic exercises are used for cardiac rehabilitation. We aimed to analyze the effects of an eight-week home-based phase II cardiac rehabilitation program performed in VR on depression and quality of life (QOL) in community-dwelling individuals who underwent open-heart surgery.

**Methods:**

A randomized controlled trial was conducted, and 49 patients who underwent elective open-heart surgery were assigned to either the VR group (n=24) or the control group. Members of the control group received a paper booklet (n=25) during phase II cardiac rehabilitation. Depression and SF-36 scores were assessed at baseline and at eight weeks.

**Results:**

The VR group showed a significant decrease in depression after eight weeks, compared to the control group (D-1.75, p=.003, n2p=0.175). The SF-36 showed no significant differences between the VR and control groups.

**Conclusions:**

A home-based VR exercise program has applications in phase II cardiac rehabilitation for open-heart surgery by reducing depression but not QOL. Therefore, integrating VR exercise into cardiac rehabilitation programs may offer a novel strategy to address mental health challenges.

## Introduction

Cardiac rehabilitation is an essential component in patient management during the recovery from cardiovascular events, and exercise-based cardiac rehabilitation is recommended for cardiac patients to improve physical, psychological, and social functioning. Phase II cardiac rehabilitation, which typically follows the initial hospital phase, involves a structured outpatient programme that is designed to enhance cardiovascular health through supervised exercise, education, and lifestyle modification (Taylor et al., 2022; Winnige et al., 2021). Although exercise is crucial for patients with cardiovascular disease and those who have undergone cardiac surgery, phase II cardiac rehabilitation remains significantly underutilized due to limited referrals and low participation rates (Mampuya, 2012; Parker & Adams, 2008; Taylor et al., 2022). After open-heart surgery, patients require individualised physical activity guidelines tailored to their exercise capacity (Parker & Adams, 2008). Evidence from a meta-analysis and systematic review has demonstrated that cardiac rehabilitation programmes delivered in hospital-, community-, or home-based settings can reduce mortality and hospitalisation rates and improve quality of life (QOL) in patients undergoing coronary artery bypass grafting (CABG) or percutaneous coronary intervention (Dibben et al., 2021). However, participation in cardiac rehabilitation programmes remains low, with only 10–30% of eligible patients engaging globally (Ramachandran et al., 2022). Furthermore, factors that influence poor cardiac rehabilitation adherence include living far from the cardiac rehabilitation facilities, a lack of transportation, financial constraints, and distance or travel time (Chindhy et al., 2020; Sugiharto et al., 2023). Therefore, these limitations led participants to undergo heart surgery and avoid rehabilitation after hospital discharge.

Mental health is another significant determinant of the overall recovery and QOL in patients with cardiac disease (Sakamoto et al., 2022). Depression and poor QOL have been reported in patients undergoing cardiac surgery to have a negative impact on recovery outcomes. Preoperative depression affects 20−47% of patients, while postoperative depression can impact 23-61% of patients, depending on the detection methods and follow-up protocols used ​(Vu & Smith, 2023). Given the high prevalence and significant impact of depression in patients undergoing cardiac surgery, ongoing research and improved clinical practices are crucial to address this critical aspect of patient care​.

In response to these challenges, virtual reality (VR) technology has emerged as an innovative tool in various medical fields and offers immersive and interactive experiences that can potentially transform conventional therapeutic approaches. Regarding the benefits of exercise, VR has shown promise in enhancing motivation, adherence, and enjoyment, which are critical for sustained engagement in physical activity. Moreover, VR exercise interventions have been reported to provide significant psychological benefits, including reductions in anxiety and depression, improvements in mood, and enhanced cognitive function (Qian et al., 2020; Wang, 2024). Therefore, VR-based exercise has had a potentially positive impact on rehabilitative physiological and psychological outcomes e.g. balance, muscle function, muscle strength, depression and QOL. Nevertheless, current telehealth and VR interventions often lack real-time physiological monitoring and individualised exercise intensity prescriptions, which are key elements for patients in Phase II cardiac rehabilitation after open-heart surgery.

**Figure 1.**
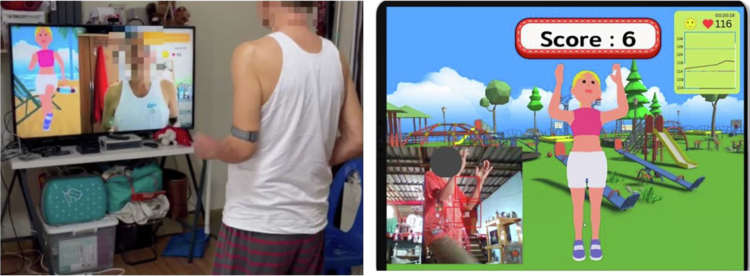
VR exercise with pulse rate monitoring.

To address these gaps, we developed a VR-based aerobic exercise programme with real-time pulse rate monitoring that can help increase physical activity in healthy individuals (Figure 1) (Yuenyongchaiwat et al., 2024). This study aimed to explore the effect of VR-based aerobic exercise on the psychological health and QOL of patients undergoing heart surgery for phase II cardiac rehabilitation.

## Material and methods

Patients who underwent open-heart surgery were invited to participate in phase II **c**ardiac rehabilitation. The medium effect size was set at 0.25, as defined by Cohen, with a statistical power of 0.90, and alpha was 0.05. The study was selected because a moderate effect was considered a realistic and conservative estimate for changes in psychological outcomes. With a statistical power of 0.90 and an alpha level of 0.05, the required sample size was determined accordingly. The required sample size for a repeated measures ANOVA with within-between interaction is calculated by using the G-power 3.1.9.4 programme. Therefore, we targeted a study cohort of 46 participants. However, since a 10% dropout rate was anticipated, 50 participants were recruited in total. The inclusion criteria were that the participants aged over 18 years, had undergone open-heart surgery after a three-week discharge from the hospital, and had no history of depression. The exclusion criteria were unstable angina within the last three months, uncontrolled sinus tachycardia, uncontrolled resting systolic blood pressure (greater than 180 mmHg), or diastolic blood pressure greater than 120 mmHg (Whelton et al., 2018). Participants were randomly assigned to either the intervention or control group at the beginning of the programme. However, the simultaneous recruitment of all participants was not feasible; therefore, randomisation was performed individually as each participant enroled. A computer-generated random sequence with a 1:1 allocation ratio was used, and assignment was carried out by an independent researcher who was not involved in recruitment or outcome assessment.

Initially, 50 participants were randomised (25 per group). Three participants (one in the intervention group and two in the control group) did not complete the baseline questionnaire and were therefore excluded, resulting in a final sample of 47 participants for analysis.

The study cohort was randomised into two groups through simple randomised sampling: by a computer programme. Baseline assessments were conducted prior to randomisation. Participants were then individually randomised to either the intervention or control group at the start of the programme. Because participants could not be recruited simultaneously, simple randomisation was performed sequentially using a computer-generated random sequence with a 1:1 allocation ratio. The experimental group received VR exercise for eight weeks, and the control group (CG) received an exercise brochure. The experimental group received a VR exercise that was prescribed by 40%–59% of heart rate reserve (HRR) or rate of perceived exertion: Borg Scale score of less than 13 out of 20. The control group received an exercise prescription brochure, which reflects standard care for patients undergoing Phase II cardiac rehabilitation in our clinical setting. The brochure provided structured guidance on aerobic exercise, including recommended frequency, intensity, and duration, consistent with established cardiac rehabilitation guidelines. This control condition ensured that all participants received an evidence-based exercise prescription. The exercise prescription brochure provided to the control group reflected usual care and standard practice for Phase II cardiac rehabilitation in the clinical setting.

Outcome measures were assessed at two timepoints: baseline (pre-intervention) and after completion of the 8-week programme (post-intervention). The protocol for the VR-based aerobic exercise programme was approved by two experts in cardiopulmonary physiotherapy and one in physical rehabilitation medicine. Table 1 presents an overview of the aerobic exercise programme.

**Table 1.** Protocol VR exercise in cardiac rehabilitation phase II.

Breathing exercise (3 mins)	10 reps/set, 3 sets
**Upper limbs exercise/ Active chest trunk mobilization (9 mins)** 1. Shoulder flexion (AP chest wall mobilization) 2. Shoulder abduction 3. Shoulder flexion to the opposite side (Posterolateral chest wall mobilization) 4. Lateral chest wall mobilization	10 reps/set, 3 sets 10 reps/set, 3 sets 10 reps/set, 3 sets 10 reps/set, 3 sets
**Lower limbs exercise (4.33 mins/cycle, total 7 cycle = 30 mins)** 1. Marching 2. Hip abduction-adduction 3. Mini squat 4. Hip-knee flexion 5. Hip flexion with adduction	F: 2-3 days/week, 8 weeks I: 40−60% heart rate reserve T: 30 mins T: Aerobic exercise
**Cool down:** Marching	5 mins
**UEs exercise/Active chest trunk mobilisation (9 mins)** 1. Shoulder flexion (AP chest wall mobilization) 2. Shoulder abduction 3. Shoulder flexion to the opposite side (Posterolateral chest wall mobilization) 4. Lateral chest wall mobilisation	10 reps/set, 3 sets 10 reps/set, 3 sets 10 reps/set, 3 sets 10 reps/set, 3 sets
**Breathing exercise (3 mins)**	10 reps/set, 3 sets

The intervention group participated in a VR exercise programme twice a week for eight weeks, whereas the CG received a prescription brochure that described standard care and the advantages of exercise and aerobic exercise. Blinded assessors (therapists who were blinded to participants’ group, i.e. VR or CG) evaluated the participants in both groups eight weeks after training completion. A physical therapist supervised the assessments of the individual's safety and monitored pulse rate, blood pressure, SpO_2_, ratings of perceived exertion, and signs and symptoms of exertional intolerance (chest pain, leg fatigue, and dizziness) by subjective examination before, during, at the end, and 5 min after the test. Furthermore, the subsequent assessment began only when hemodynamic variables returned to their resting levels.

Thai version 36-Item Short Form Survey (Thai SF-36) was used to assess QOL. The SF-36 has been validated and translated into various languages and cultures, has good reliability and validity, and is divided into two main parts: physical health and mental health components (Krittayaphong et al., 2000; Leurmamkul & Paranee, 2005). The SF-36 covers eight components in total: physical functioning, physical functioning, bodily pain, general health, vitality, social functioning, role emotion, and mental health. The physical dimension is represented by the Physical Component Summary (PCS), and the mental dimension is represented by the Mental Component Summary (MCS). The Patient Health Questionnaire-9 (PHQ-9) is self-administered and assesses depression over the most recent two weeks. The Thai version of the PHQ-9 was translated by Lotrakul et al. and displays a sensitivity level of 0.84 and a specificity of 0.77 (Lotrakul et al., 2008). All participants were asked to complete the SF-36 and Thai PHQ-9 before and after the eight-week intervention programme.

The intention-to-treat analysis was conducted for participants in both VR and CG who were dropped or did not complete the outcome measure. Missing post-intervention data were addressed using the Last Observation Carried Forward (LOCF) method, whereby the pre-intervention score was substituted for the missing value. A two-way mixed repeated ANOVA (time (2) × type (2)) was used to compare depression and QOL between and within groups. Statistical significance was set at *p* < 0.05.

## Results

**Figure 2.**
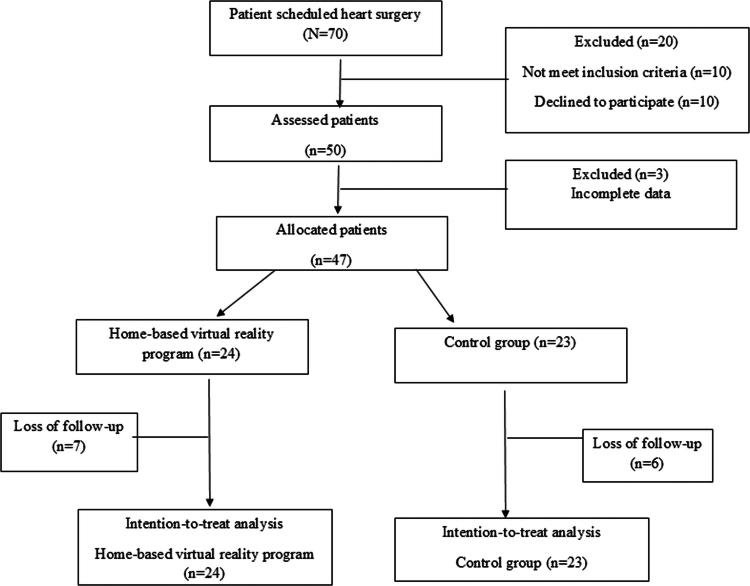
Recruitment of participant.

Fifty participants were enroled in the study; however, three did not complete the questionnaires (SF-36 and PHQ-9). Therefore, 47 individuals were enroled in the baseline assessment (VR = 24, CG = 23). However, after an 8-week intervention programme, seven participants in the VR and six in CG had not completed the study due to unavailability. Therefore, the intention-to-treat analysis was conducted with 47 participants included in the final analysis (Figure 2).

The average participant was an older adult with a mean age of 60.91 ± 11.56 years. Most participants were males (76.60%), and over half of the 47 participants had CABG (61.70%). Additionally, half of the participants had a history of dyslipidemia (51.06%) and hypertension (48.94%). The data characteristics of the participants who underwent open-heart surgery in phase II revealed no significant differences between the VR and CG cohorts, with the exception of sex (see Table 2). The most common comorbidities were dyslipidemia (51.06%), hypertension (48.94%), and diabetes mellitus (25.53%).

**Table 2.** Characteristics of participants.

	Total (*N* = 47) Mean ± SD	VRG (*N* = 24) Mean ± SD	CG (*N* = 23) Mean ± SD	t(test)/X^2^	*p*-value
Age (years)	60.94 ± 11.40	63.04 ± 7.99	58.74 ± 13.97	1.303	.199
BMI (kg/m^2^)	23.82 ± 4.52	24.67 ± 4.10	22.94 ± 4.85	1.323	.193
**Sex**					
Male (%)	36 (76.60)	15 (62.50)	21 (91.30)	5.436	.020
Female	11 (23.40)	9 (37.50)	2 (8.70)		
**Types of operation**				4.577	.101
CABG	29 (61.70)	16 (66.67)	13 (56.52)		
Valve surgery	14 (29.79)	8 (33.33)	6 (26.09)		
Combined CABG and valvular	4 (8.51)	0 (0.00)	4 (17.39)		
**Comorbidities**					
Diabetes mellitus				1.570	.210
Diabetes mellitus	12 (25.53)	8 (33.33)	4 (17.39)		
No history of diabetes mellitus	35 (74.47)	16 (66.67)	19 (82.61)		
Hypertension				0.189	.664
Hypertension	23 (48.94)	11 (45.83)	12 (52.17)		
No history of hypertension	24 (51.06)	13 (54.17)	11 (47.83)		
Dyslipidemia				1.733	.188
Dyslipidemia	24 (51.06)	10 (41.67)	14 (58.33)		
No history of dyslipidemia	23 (48.94)	14 (60.87)	9 (39.13)		
COPD/Asthma				0.979	.322
COPD/Asthma	1 (2.13)	1 (4.17)	0 (0.00)		
No history of COPD/Asthma	46 (97.87)	23 (95.83)	23 (100.00)		

CABG: coronary artery bypass graphing, COPD: chronic obstructive pulmonary disease, VRG: virtual reality group, CG: control group.

A component summary of SF-36 scores (physical and mental components) and depression scores is shown in Table 3. After eight weeks, the study identified improvement in the physical components of participants in both VR and CG cohorts (Δ 8.35 ± 2.38 and Δ 7.57 ± 2.43, respectively). In addition, significantly decreased depression was observed in the VR group after the eight-week intervention programme (∆–1.75, *p* = .003, n^2^_p_ = 0.175), but not in the CG (∆ 1.04, *p* = .078). In addition, compared to the CG, a significant reduction in depression was observed in the VR group (∆–2.88, *p* = .049).

**Table 3.** Quality of life and depression scores before and after 8-week VR exercise.

	VRG (*n* = 24)	CG (*n* = 23)	Mean difference (SE) VR-control	*p*-value between groups
**SF-36 (Physical component scales)**				
Before mean ± SD	37.26 ± 8.41	39.50 ± 12.88	−2.241 ± 3.16	.482
After mean ± SD	45.60 ± 11.11	47.07 ± 10.85	−1.465 ± 3.21	.650
Mean difference (after-before) ± SE	8.35 ± 2.38	7.57 ± 2.43		
*p* value within group	.001	.003		
**SF-36 (Mental component scales)**				
Before mean ± SD	49.98 ± 14.54	50.32 ± 14.17	−0.332 ± 4.19	.937
After mean ± SD	53.70 ± 10.66	48.28 ± 15.24	5.420 ± 3.82	.163
Mean difference (after-before) ± SE	3.716 ± 2.86	−2.036 ± 2.92		
*p* value within group	.201	.490		
**Depression**				
Before mean ± SD	4.92 ± 4.91	5.00 ± 5.05	−0.08 ± 1.45	.954
After mean ± SD	3.17 ± 3.67	6.04 ± 5.86	−2.88 ± 1.42	.049
Mean difference (after-before) ± SE	−1.75 ± 0.57	1.04 ± 0.58		
*p* value within group	.003	.078		

VRG: virtual reality group, CG: control group.

Eight dimensions of the SF-36 were analysed: physical (i.e. physical functioning, physical role limitations, bodily pain, and general health perceptions) and mental component (i.e. energy/vitality, social functioning, emotional role limitations, and mental health) summaries; see Table 4. Regarding the physical component domains, VR and CG significantly increased physical functioning (Δ 4.13 and Δ 1.83, respectively) and role physical scores (Δ 1.58 and Δ 1.17, respectively) after the eight-week intervention programme. However, no statistically significant difference was observed between the VR group and CG (*p* > .05).

**Table 4.** Subgroup analysis of quality of life before and after 8-week VR exercise.

	VRG (*n* = 24)	CG (*n* = 23)	Mean difference (SE) VR-control	*p* value between groups
**Physical functioning (scales)**				
Before mean ± SD	22.00 ± 4.42	23.52 ± 4.75	−1.52 ± 1.34	.261
After mean ± SD	26.13 ± 3.64	25.35 ± 3.92	0.78 ± 1.10	.484
Mean difference (after-before) ± SE	4.13 ± 0.87	1.83 ± 0.89		
*p* value within group	<.001	.046		
**Role-Physical (scales)**				
Before mean ± SD	5.17 ± 1.74	5.57 ± 2.00	−0.40 ± 0.55	.468
After mean ± SD	6.75 ± 1.73	6.74 ± 1.76	0.01 ± 0.51	.983
Mean difference (after-before) ± SE	1.58 ± 0.38	1.17 ± 0.39		
*p* value within group	<.001	.004		
**Bodily pain (scales)**				
Before mean ± SD	9.41 ± 1.51	9.49 ± 1.62	−0.07 ± 0.46	.871
After mean ± SD	9.17 ± 1.89	9.24 ± 2.20	−0.08 ± 0.60	.898
Mean difference (after-before) ± SE	−0.25 ± 0.27	−0.24 ± 0.28		
*p* value within group	.366	.380		
**General health (scales)**				
Before mean ± SD	16.88 ± 3.00	15.54 ± 2.60	1.34 ± 0.82	.111
After mean ± SD	16.59 ± 2.58	15.60 ± 2.63	0.99 ± 0.76	.199
Mean difference (after-before) ± SE	−0.28 ± 0.57	0.06 ± 0.58		
*p* value within group	.620	.917		
**Vitality (scales)**				
Before mean ± SD	16.25 ± 3.23	17.22 ± 4.31	−0.97 ± 1.11	.387
After mean ± SD	18.96 ± 2.91	17.43 ± 3.13	1.52 ± 0.88	.091
Mean difference (after-before) ± SE	2.71 ± 0.66	0.22 ± 0.67		
*p* value within group	<.001	.749		
**Social functioning (scales)**				
Before mean ± SD	9.08 ± 1.56	8.87 ± 2.22	0.21 ± 0.56	.703
After mean ± SD	9.46 ± 1.10	9.87 ± 2.34	−0.41 ± 0.53	.442
Mean difference (after-before) ± SE	0.38 ± 0.46	1.00 ± 0.47		
*p* value within group	.463	.040		
**Role-emotional (scales)**				
Before mean ± SD	5.33 ± 1.20	5.48 ± 1.16	−0.15 ± 0.35	.677
After mean ± SD	5.50 ± 1.14	5.48 ± 1.16	0.02 ± 0.34	.949
Mean difference (after-before) ± SE	0.17 ± 0.20	0.00 ± 0.21		
*p* value within group	.410	1.00		
**Mental health (scales)**				
Before mean ± SD	25.17 ± 4.29	25.70 ± 3.62	−0.53 ± 1.16	.651
After mean ± SD	26.54 ± 3.73	25.26 ± 3.56	1.28 ± 1.06	.235
Mean difference (after-before) ± SE	1.38 ± 0.66	−0.44 ± 0.67		
*p* value within group	.042	.521		

VRG: virtual reality group, CG: control group.

According to the mental component domains, increasing vitality and mental health scales were noted in the VR group (Δ2.71 and Δ1.38, respectively), whereas social function scales improved in the CG (Δ 1.00). However, no statistically significant differences in improvement were identified between the VR and CG cohorts after the eight-week intervention period (*p* > .05).

## Discussion

This study investigated effects of VR exercise on depression and QOL in patients who underwent open-heart surgery during phase II cardiac rehabilitation. Twenty-four participants in the VR group and 23 in the CG were evaluated using an intention-to-treat analysis. The study revealed decreased depression in the VR group after an eight-week intervention, compared to the CG. In addition, improvements in vitality and mental health scales were noted in the VR exercise programme after eight weeks, but not in the control group. Both the VR and control groups showed significant differences in the physical components before and after the intervention, but not between the groups.

In a previous study, the QOL of patients following surgery was shown to be strongly influenced by impaired mental and physical conditions that were associated with decreased aerobic fitness (Pinto et al., 2016). Moreover, individuals could have decreased QOL after surgery, in addition to functional limitations. Such conditions can result from the absence of movement or physical inactivity due to psychological and/or physiological effects (Kronzer et al., 2017).

QOL in this study was assessed using the Thai SF-36. The results were divided into two main categories: mental and physical health. Notably, the effectiveness of VR on psychological health and QOL in patients with cardiac rehabilitation has been noted recently (Bashir et al., 2023; Chen et al., 2022; García-Bravo et al., 2020; Micheluzzi et al., 2024; Vieira et al., 2018). The present study revealed an improvement in the SF-36 in the physical component, that is, physical functioning and role-physical domains, after eight-week interventions in both groups, with no statistically significant differences between the two groups. The improvement in PCS in both groups may have resulted from physical recovery following cardiac surgery (Archer et al., 2019), which could help enhance an individual’s perception of their daily life and ability to work (Barnason et al., 2008). Therefore, no statistically significant increase was observed in either the group’s general health or their body pain.

Regarding the mental component, participants who performed VR showed improvements in mental health and vitality domains, but not in social function and role emotion. This can be explained by the ceiling effect, in which all participants showed high scores for social function and role emotion in both the CG and the VR group. Similar to the findings of our study, Garcia-Bravo et al. reported that, in patients with ischaemic heart disease, using VR exercise for eight weeks improved QOL in physical, mental components, and vitality subscales (García-Bravo et al., 2020). Moreover, Jóźwik et al. reported that participants in VR showed statistically significant improvements in mental health, by reducing stress, anxiety, and depression (Jóźwik et al., 2021). Additionally, VR in **c**ardiac rehabilitation has also been shown in a systematic study to improve QOL by reducing stress, fatigue, and depression (Bouraghi et al., 2023). Engagement of the participants in multi-sensory learning was achieved by using a combination of moving images, sound, and text. The augmented reality could improve mental health components in patients who undergoing CABG through the use of innovative technology, like VDO physical exercise. The process improved self-efficacy, which is associated with improved mental health through improved adherence to exercise (Ghlichi Moghaddam et al., 2023). In particular, the VR exercise displays the feature of automatic speed adjustment, which allows participants to continuously perform exercises at the target heart rate (40–59% HRR) through real-time monitoring of pulse rate and dyspnoea score, which helps users feel safe and confident while exercising. Furthermore, the exercise protocol of this VR programme was a moderate-intensity exercise, which can be considered an appropriate level of intensity, not too exhausting to cause discomfort, and suitable for improving emotional and mental states. Accordingly, the use of moderate aerobic VR exercises may increase exercise motivation, thus resulting in improved functional capacity and QOL. Consequently, the VR exercise programme was developed to reduce limitations and barriers to exercise during **c**ardiac rehabilitation by designing a suit for functional movement, which involved preparation of the body for activities performed in daily life after surgery. Thus, improved mental health can attained in participants who perform VR aerobic exercise programmes.

Participants who engaged in VR had lower depression scores. Regarding a systematic review and meta-analysis, Chen reported positive QOL and psychological outcomes in 10 studies with randomised control trials, along with reduced depression during **c**ardiac rehabilitation (Chen et al., 2022). Furthermore, Bashir et al. conducted seven studies with 747 **c**ardiac rehabilitation participants and showed a reduction in anxiety but not in functional capacity through the use of VR (Bashir et al., 2023). Therefore, the advantage VR exercise programmes involve its ability to stimulate a psychophysical state that is marked by decreased negative emotions and stress and heightened positive emotions. Ghlichi Moghaddam et al. reported that the use of innovative technology, such as VDO physical exercise, improved self-efficacy, which was associated with improved mental health through improved exercise adherence (Ghlichi Moghaddam et al., 2023). VR technology also improves negative psychological effects related to phobias and post-traumatic stress disorder. Consequently, individuals who have undergone cardiac surgery may benefit from VR by experiencing less stress and more enjoyment. Moreover, VR during **c**ardiac rehabilitation has been shown in a systematic study to improve QOL by reducing stress, fatigue, and depression (Bouraghi et al., 2023). VR exercise could contribute to improving motivation or adherence to exercise, whereas participants in the CG tended to present a significantly lower mental component. Notably, the use of VR exercise helps to improve mental health in patients who have undergone phase II open-heart surgery.

A significant limitation of the study that should be noted when interpreting our findings was the enrichment of male participants, because the rate of CABG surgery is higher in males than in females (Nurkkala et al., 2022). Thus, a sex bias could affect the generalisability of our findings. There is also the potential for population or external validity bias due to most participants having undergone CABG; therefore, the results of this study should not be generalised to other conditions in patients with open-heart surgery without further follow-up investigations. Another limitation of this study is the relatively small sample size, which may have resulted in an imbalance of baseline characteristics, such as sex distribution, between the randomised groups. This may affect the generalisability and interpretation of the findings. Furthermore, the study did not record characteristics, such as ejection fraction, New York Heart Association (NYHA) functional class, laboratory data, or physical activity levels. Most of the participants in this study were NYHA classes 1–2, which means that they had no symptoms, no limitations in ordinary physical activity, nor mild shortness of breath during ordinary physical activity. Furthermore, these results might not be representative nor applicable to patients with NYHA classes 3 and 4. Notably, physical activity in both groups and exercise in the control group were not recorded, which were confounding factors in this study. Another limitation was that the use of VR technology was limited by many factors, such as education, age, economic status, and digital literacy. Finally, although both groups received comparable exercise prescriptions, the intervention and control conditions differed in their mode of delivery. The VR-based intervention provided an interactive experience, whereas the control group relied on a self-guided exercise brochure. These differences in participant engagement may have contributed to nonspecific or placebo effects, such as increased motivation or expectancy, in the intervention group. Future studies should consider using active control condition to enhance comparability between groups.

## Conclusion

The VR exercise programme reported herein reduced depression and quality of life in patients after phase II open-heart surgery. In addition, participants in VR had a significant decrease compared with the control group. Therefore, the beneficial effect of VR exercise programmes is a reduction in the depression scale in patients following phase II open-heart surgery.

## Data Availability

The data supporting this study's findings are available from the corresponding author upon reasonable request.

## Registration number

Thai Clinical Trial Registry (TCTR) TCTR20230602001.
